# Comment on “Pain Perception of the First Eye versus the Second Eye during Phacoemulsification under Local Anesthesia for Patients Going through Cataract Surgery: A Systematic Review and Meta-Analysis”

**DOI:** 10.1155/2020/7364767

**Published:** 2020-07-20

**Authors:** Liang Sun, Haiyan An, Yi Feng

**Affiliations:** Department of Anesthesiology, Peking University People's Hospital, Beijing 100044, China

With much interest, we read the paper entitled “Pain Perception of the First Eye versus the Second Eye during Phacoemulsification under Local Anesthesia for Patients Going through Cataract Surgery: A Systematic Review and Meta-Analysis” [1]. This topic is of heightened interest to ophthalmologists, anesthesiologists, as well as relevant health professionals providing care for patients undergoing cataract surgery. From the perspectives of anesthesiologists, we would like to express some opinions and share some experience on the pain management in the setting of cataract surgery [2].

First, one crucial finding of this study is that the pain scores of the first eye shortly after surgery under local anesthesia were significantly lower compared with the second eye, and the authors ascribed it to lower anxiety before the second surgery. This explanation was really interesting, though the pooled analysis comparing the anxiety scores retrieved only two studies with relatively small samples. However, if this is the case, it suggests that sedation of patients may play a very crucial role on patient's perception of pain [3]. Taking into account the importance of sedation and analgesia, we reviewed the enrolled studies in this meta-analysis and found that patients in 2 of 8 studies received sedatives and/or analgesics. Herein, we suggest the authors could further provide the detailed information of sedatives and analgesics administered to the patients and thus perform subgroup analysis based on these two confounding factors. Therefore, the limitations mentioned above prevent drawing concrete conclusions from the present study.

Second, we should temporarily move the eye away from the limitations of the current study. Actually, this study urged us to rethink the initiation of sedation and analgesia in patients undergoing cataract surgery. In fact, sedation is routinely administered for cataract surgery in some regions of the world, whereas other countries have very low rates of sedation, or none at all, considering their potential side effects (e.g., respiratory depression) [4]. Similarly, we observed that patients in most of the included studies of this meta-analysis did not accept any sedatives and/or analgesics. To the best of our knowledge (especially, the anesthesiologists), sedatives can increase the threshold of pain perception of patients and thus strengthen the potency of analgesia [5].

In our single center, we, anesthesiologists, seldom encountered such complaints from the ophthalmologists and patients. Department of Anesthesiology in our hospital really emphasizes the importance of sedation and analgesia in the setting of cataract surgery. Apart from the local anesthesia, midazolam (0.03 mg/kg) and fentanyl (50 *μ*g) are routinely administered to the patients when there is no contraindication, and we will make proper reduction of sedatives and analgesics according to the age and concomitant complications of the patients. Simultaneously, nonsteroidal anti-inflammatory drugs (NSAIDs) will be prescribed if indicated. Certainly, all of the cataract surgeries are under monitored anesthesia care (MAC) for safety concern. Additionally, music with a soft and meditative serenity is routinely played in the operating room [6]. After carrying out this bundled care of sedation, analgesia, and music, the patients can cooperate with the surgery well and seldom complain the pain during the perioperative period. To date, it almost became the guideline of cataract surgery with mode of multidisciplinary team (MDT) management on sedation and analgesia, which involves the ophthalmologists, anesthesiologists, nurses, as well as other health-associated professionals together dedicating to the enhanced recovery after surgery (ERAS) of the patients, and the outcomes of patients were very satisfied.

Finally, we thank Shi et al. [1] for their innovative work on this study and made us rethink sedation and analgesia in cataract surgery, as well as exploring more promising strategies to the perioperative pain management in the ERAS era.
